# Overexpression of miR-584-5p inhibits proliferation and induces apoptosis by targeting WW domain-containing E3 ubiquitin protein ligase 1 in gastric cancer

**DOI:** 10.1186/s13046-017-0532-2

**Published:** 2017-04-21

**Authors:** Qing Li, Zheng Li, Song Wei, Weizhi Wang, Zheng Chen, Lei Zhang, Liang Chen, Bowen Li, Guangli Sun, Jianghao Xu, Qiang Li, Lu Wang, Zhipeng Xu, Yiwen Xia, Diancai Zhang, Hao Xu, Zekuan Xu

**Affiliations:** 1 Department of General Surgery, The First Affiliated Hospital of Nanjing Medical University, No.300, Guangzhou road, Nanjing, Jiangsu province China; 2 Department of Surgery, Vanderbilt University Medical Center, Nashville, Tennessee USA

**Keywords:** miR-584-5p, WWP1, Proliferation, Apoptosis, Cellular senescence, TGFβ signaling pathway, Gastric cancer

## Abstract

**Background:**

MicroRNAs are endogenously expressed, small non-coding RNAs that modulate gene expression by targeting specific mRNAs, resulting in translational repression or mRNA degradation. Although miR-584-5p has been reported to play a vital role in various malignancies, its role and the molecular mechanisms underlying the effects of miR-584-5p in gastric cancer (GC) remain to be clarified. In this study, we investigated the role of miR-584-5p in GC.

**Methods:**

The expression of miR-584-5p and its specific target gene were determined in human GC specimens and cell lines by microRNA real-time polymerase chain reaction (RT-PCR), quantitative RT-PCR (qRT-PCR) and Western blot. The effects of miR-584-5p depletion or ectopic expression on GC proliferation were evaluated in vitro using CCK-8 proliferation assays, 5-ethynyl-2'-deoxyuridine (EdU) incorporation, colony formation assays and cell-cycle assays and the in vivo effects were investigated using a mouse tumorigenicity model. Cell apoptosis was evaluated by in vitro flow cytometric analysis, cell viability assays and in vivo TUNEL assays. Luciferase reporter assays were employed to identify interactions between miR-584-5p and its specific target gene.

**Results:**

A series of in vitro and in vivo gain- and loss-of-function assays revealed that miR-584-5p inhibited GC cell proliferation, while apoptosis was induced. Luciferase reporter assays and Western blot analysis revealed WWP1 to be a direct target of miR-584-5p. The effects of miR-584-5p-mimic were rescued by WWP1 overexpression. In contrast, the effects of the miR-584-5p-inhibitor were impaired by WWP1-shRNA. Furthermore, miR-584-5p expression levels correlated negatively with WWP1 protein expression in GC tissues and GC cell lines. A series of investigations indicated that miR-584-5p promoted senescence and activated the TGFβ signaling pathway by downregulation of WWP1.

**Conclusion:**

Taken together, these results suggest that downregulation of miR-584-5p contributes to tumor progression by downregulation of WWP1, thus, highlighting the potential of miR-584-5p as a therapeutic target for human GC.

## Background

Gastric cancer (GC) is one of the most prevalent lethal malignancies worldwide, particularly in Eastern Asia [1]. Despite evident advances in the treatment of early GC, including surgical techniques, radiochemotherapy, adjuvant therapy, molecular targeted therapy and earlier diagnosis, the 5-year survival rate for advanced GC remains only 5%–20% and the median overall survival for advanced GC is less than 1 year [2, 3]. Hence, clarification of the molecular mechanisms relevant to GC development and progression is urgently required.

MicroRNAs (miRNAs) are endogenously expressed, small, non-coding RNAs consisting of 20–24 nucleotides [4]. MiRNAs downregulate gene expression by binding to the 3′-untranslated regions (3′-UTR) of specific target messenger RNAs (mRNAs), resulting in inhibition of translation or mRNA degradation [5]. MiRNAs regulate numerous cellular processes, such as cell proliferation, apoptosis, migration, invasion and differentiation [6, 7]. Furthermore, aberrant expression of miRNAs have been reported in many cancers [8–10]. Gain- or loss-of miRNA functions contribute to the development of cancer by silencing tumor suppressors or activation of oncogenes. MiR-584-5p, which is located on chromosome 5q32, was reported to be downregulated in certain cancers, including human neuroblastoma [11], thyroid carcinoma [12], glioma [13] and human clear cell renal cell carcinoma [14]. Nonetheless, the biological functions and molecular mechanisms underlying the role of miR-584-5p in GC remain to be elucidated. In this study, we discovered that miR-584-5p was downregulated in GC and that miR-584-5p overexpression suppressed the progression of GC both in vitro and in vivo.

WW domain-containing E3 ubiquitin protein ligase 1 (WWP1) is involved in many biological processes, such as cell proliferation, apoptosis, senescence and protein trafficking [15]. WWP1 delays cellular senescence and negatively regulates the TGFβ signaling pathway, which regulates cell proliferation, differentiation, apoptosis, and migration in numerous cell types [16, 17]. Furthermore, it has been reported that WWP1 acts as an oncogenic factor in GC [18]. In the present study, we used bioinformatic prediction and experimental confirmation studies to demonstrate that WWP1 plays a pivotal role in human GC and is a putative direct target of miR-584-5p.

Therefore, we investigated the role of miR-584-5p in GC and its relationship with WWP1. Our results indicated that miR-584-5p downregulation is vital for the development and progression of GC, highlighting the potential of miR-584-5p as a therapeutic target in GC.

## Methods

### Tissue specimens

We collected paired tumorous and adjacent non-tumorous human gastric tissues from 75 patients with GC who underwent radical gastrectomy at the Department of General Surgery, First Affiliated Hospital, Nanjing Medical University, China. Written informed consent was obtained from the patients or their relatives before specimen collection. This study was approved by the Institutional Ethical Board of the First Affiliated Hospital of Nanjing Medical University. After resection and prior to RNA and protein extraction, tissue samples were frozen and stored in liquid nitrogen. Histopathological diagnoses and grading was carried out by two experienced pathologists.

### GC cell lines

The human GC cell lines, MGC803, AGS, BGC823, HGC27 and SGC7901 as well as the normal human gastric epithelial cell line GES-1 were purchased from the Cell Center of Shanghai Institutes for Biological Sciences. Cells were cultured in RPMI-1640 medium supplemented with 10% fetal bovine serum (FBS; WISENT, Canada) and antibiotics (1% penicillin/streptomycin, Gibco, USA) at 37 °C in a humidified atmosphere under 5% CO2.

### Quantitative real-time polymerase chain reaction(qRT-PCR)

Total RNA was extracted from GC tissues and cells with the TRIzol reagent (Invitrogen) according to the manufacturer’s instructions, and was reverse transcribed into cDNA using PrimeScript RT Reagent (TaKaRa). qRT-PCR was performed using a 7500 Real-time PCR System (Applied Biosystems, Carlsbad, CA, USA) with SYBR Premix Ex Taq Kit (TaKaRa) with the following primers: WWP1 forward, 5′-TGCTTCACCAAGGTCTGATACT-3′ and WWP1 reverse, 5′-GCTGTTCCGAACCAGTTCTTTT-3′; β-actin forward, 5′-GCATCGTCACCAACTGGGAC-3′ and β-actin reverse, 5′-ACCTGGCCGTCAGGCAGCTC-3′. WWP1 expression levels were normalized to β-actin and relative expression was calculated using the 2^-ΔΔCT^ method.

### MiRNA RT–PCR

Total RNA was extracted as described previously. Target-specific reverse transcription and the TaqMan microRNA assay were carried out using the Hairpin-it™ miRNA qPCR Quantitation Kit (GenePharma, China) according to the manufacturer’s instructions. The reactions were also performed using the 7500 Real-Time PCR System with the following primers: hsa-miR-584-5p forward, 5′-TTATGGTTTGCCTGGGACTGAG-3′; Universal, 5′-GCGAGCACAGAATTAATACGAC-3′; U6 forward, 5′-CTCGCTTCGGCAGCACA-3′ and U6 reverse, 5′-AACGCTTCACGAATTTGCGT-3′. Expression of miR-584-5p was normalized to snRNA U6 and relative expression was calculated using the 2^-ΔΔCT^ method. All procedures were performed in triplicate.

### Cell proliferation and viability assays

Cell Counting Kit-8 (CCK-8; Dojindo, Kumamoto, Japan) was used to evaluate cell proliferation and viability according to the manufacturer’s instructions. For cell proliferation assays, GC cells were seeded into 96-well plates (2,000 cells/well) and cultured with RPMI 1640 (10% FBS) for 5 days. CCK-8 solution (10 μl) was added to each well at the indicated time-point and cells were incubated for 2 h at 37 °C. For detection of cell viability, cells were seeded into 96-well plates (5,000 cells/well) and incubated for 6 h to allow static adherence for reducing the effects of proliferation resulting from alterations in miR-584-5p expression. Cells were incubated in RPMI-1640 medium without FBS for 48 h as described previously [19]. Cell proliferation and viability were assessed by measurement of the optical density measured at 450 nm.

### Colony formation assay

Four groups of stable GC cells were plated in 6-well plates (400 cells/well) and cultured in RMPI-1640 medium for 3 weeks. Proliferating colonies were stained with crystal violet and colonies consisting of 50 cells or more were counted and photographed for statistical analysis. All procedures were performed in triplicate.

### 5-Ethynyl-2’-deoxyuridine (EdU) assay

Cell proliferation was measured using the EdU assay kit (RiboBio, China). Briefly, cells were seeded into 24-well plates (2 × 10^4^ cells/well) and cultured with RPMI 1640 (10% FBS) for 24 h before the addition of EdU (50 μM). Cells were then incubated for 2 h at 37 °C, fixed in 4% formaldehyde for 30 min and permeabilized with 0.5% TritonX-100 for 10 min at room temperature. After washing with PBS, 1× ApolloR reaction cocktail (400 μl) was added to react with the EdU for 30 min. Subsequently, Hoechest33342 (400 μl) was added for 30 min to visualize the nuclei. Images of cells were obtained under a Nikon microscope (Nikon, Japan). Proliferation was analyzed using the mean number cells in three fields for each sample.

### Flow cytometric analysis of cell-cycle and apoptosis

Cell-cycle and apoptosis were analyzed using flow cytometric (FCM) methods. Transfected cells were digested with trypsin and centrifuged at 1,200 rpm for 5 min. Cells were washed carefully with PBS (twice) and fixed in 75% ethanol prior to storage at -20 °C overnight. Before FCM detection, the cells were washed twice with PBS, incubated with RNAse, and stained with PI staining solution (500 μl) for 15 min at room temporature for cell-cycle analysis. Apoptotic cells were stained with PI (10 μg/ml; Sigma) and Annexin V-FITC (50 μg/ml, BD) in the dark for 15 min at room temperature, according to the manufacturer’s instructions. Data were acquired on a FACScan flow cytometer (BD, Franklin Lakes, NJ, USA).

### Western blot analysis

The proteins extracted from GC cells and tissues were resolved by sodium dodecyl sulfate polyacrylamide gel electrophoresis (SDS-PAGE) and transferred to a polyvinylidene fluoride (PVDF) membrane. After blocking with 5% non-fat powdered milk in Tris-buffered saline for 2 h, membranes were incubated at 4 °C overnight with the following specific primary antibodies: WWP1 (Sigma, 1:200), smad4, TGFβR (Cell Signaling Technology, 1:1,000), smad2, p15, p16, p21, p53, and GAPDH (Santa Cruz Biotechnology, 1:200). The membranes were then incubated with HRP-conjugated anti-mouse or anti-rabbit IgG (1:2,000) at room temperature for 2 h and then washed with TBST buffer three times. Protein expression levels were visualized using an enhanced chemiluminescence (ECL) detection system. GAPDH was used as an internal control.

### Vector constructs, lentivirus production and cell transfections

Commercially available lentiviral vectors were used to construct the LV2-hsa-miR-584-5p-mimic vector (miR-584-5p-mimic) and the LV2-hsa-miR-584-5p-inhibitor vector (miR-584-5p-inhibitor) (GenePharma, Shanghai, China). These constructs were verified by DNA sequencing before being used to overexpress or knockdown miR-584-5p in GC cells. The LV2 empty lentiviral construct (miR-NC) served as a negative control. The miR-584-5p-NC, miR-584-5p-mimic and miR-584-5p-inhibitor lentiviral vectors were used to at an appropriate multiplicity of infection (MOI) to infect MGC803 and SGC7901 cells grown to 40%–50% confluence. Stable cells lines were generated by selecting transfected cells in cultures containing 5 μg/ml puromycin (Sigma, Aldrich) for 5 days. Subsequently, cells were analyzed for miR-584-5p expression using the Hairpin-it™ miRNA qPCR Quantitation Kit. Vectors for the overexpression and shRNA targeting of human WWP1 using lentiviral gene transfer and containing the puromycin resistance sequence were constructed by Genepharma Biotech (Shanghai, China). The scrambled lentiviral construct was used as a negative control. MGC803 and SGC7901 cells (at 40%–50% confluence) were transfected with the lentiviral vectors (LV-WWP1, LV-NC; WWP1-shRNA, WWP1-shcontrol). Stable cells lines were generated by selecting transfected cells in cultures containing 5 μg/ml puromycin (Sigma, Aldrich) for 5 days. WWP1 expression was then analyzed by qRT-PCR and Western blot.

### 3'-UTR luciferase construct and dual luciferase reporter assays

Sequences corresponding to the 3'-UTR of WWP1 mRNA and containing the wild-type or mutated miR-584-5p binding sequence were synthesized by GeneScript (Nanjing, China). To generate the WWP1 3'-UTR reporter constructs (pGL3-WT-WWP1 and pGL3-MUT-WWP1), these sequences were cloned into the *Fse*I and *Xba*I restriction sites of the pGL3 luciferase control reporter vector (Promega, USA). MGC803 and SGC7901 cells were seeded in 24-well plates (5 × 10^5^ cells/well) and incubated for 24 h before transfection. Cells were co-transfected with 0.12 μg of either the pGL3-WT-WWP1 or pGL3-MUT-WWP1 3'-UTR reporter plasmids together with 40 nM of miR-584-5p mimic or negative control oligoribonucleotides using Lipofectamine 2000 (Invitrogen). MGC803 and SGC7901 cells were also transfected with 0.01 μg of Renilla luciferase expression plasmid as a reference control. Firefly and Renilla luciferase activities were detected using dual luciferase reporter assays (Promega, E1910, WI, USA) at 36 h post-transfection according to the manufacturer’s instruction. The relative luciferase activity was calculated according to the ratio of firefly fluorescence and Renilla fluorescence.

### Immunofluorescence microscopy

MGC803 and SGC7901 cells (2,000 cells/well) cultured on collagen-coated glass coverslips were rinsed with PBS twice before fixation with 4% formaldehyde for 20 min at room temperature. The cells were rinsed then with PBS three times and permeabilized with 0.2% Triton X-100 for 10 min. To block non-specific binding, the cells were incubated with PBS containing 1% BSA for 30 min and then incubated with the primary antibody TGFβ (Cell Signaling Technology, 1:1,000) at 4 °C. Subsequently, cells were washed again and incubated with the secondary antibody Cy™ 3-Goat Anti-Rabbit IgG(Jackson, 1:100) for 2 h before staining with DAPI for 5 min. After the final wash, fluorescence microscopy (Nikon, Japan) was used to take photomicrographs of the cells. The obtained images were merged. TGFβ was stained red and the DAPI-stained nuclei appeared blue.

### SA-β-gal staining

SA-β-gal staining was performed using the Senescence β-Galactosidase Staining Kit (Cell Signaling Technology) according to the manufacturer’s instruction. Briefly, MGC803 and SGC7901 cells were seeded in 24-well plates (2 × 10^4^ cells/well). Cells were washed twice in PBS and fixed with Fixative Solution for 15 min at room temperature. After fixation, cells were stained with 500 μl complete β-gal staining solution per well overnight at 37 °C. The complete β-gal staining solution consists of Staining Solution, Solution A, Solution B and X-gal solution (final concentration of 1 mg/ml). After staining, images of the cells were captured using a microscope(Nikon, Japan).

### In vivo tumor xenograft model

All animal experiments were conducted according to the guidelines of the Nanjing Medical University (NJMU) Institutional Animal Care and Use Committee. Twelve female BALB/c nude mice (aged 4 weeks) were purchased from the Animal Center of NJMU and were randomly allocated to four groups (*n* = 3 per group). Stably transfected cell lines (SGC7901-miR-NC, SGC7901-miR-584-5p-inhibitor, MGC803-miR-NC, and MGC803-miR-584-5p-mimic) were inoculated bilaterally and subcutaneously into the flanks of the nude mice. Bidimensional tumor measurements were taken with Vernier calipers every 4 days, and the mice were euthanized after 3 weeks. The volume of the implanted tumor was calculated using the formula: volume = (width^2^ × length)/2.

### Immunohistochemical (IHC) analysis of specimens and subcutaneous xenograft model

All specimens and implanted tumors were fixed in 4% formalin and then embedded in paraffin. After blocking endogenous peroxides and proteins, sections (thickness, 4 μm) were incubated overnight at 4 °C with primary antibodies for specific detection of WWP1 or Ki-67 (Maixin Bio, China). After washing with PBS, sections were incubated with HRP-Polymer-conjugated secondary antibody at 37 °C for 1 h. Subsequently, sections were stained with 3,3-diaminobenzidine solution for 3 min and the nuclei were counterstained with hematoxylin. Tumor sections were examined in a blinded manner. The percentage of positive tumors and cell-staining intensity was determined based on three randomly selected fields for each section.

### TUNEL assay

Implanted tumors were fixed in 4% formalin, paraffin-embedded and sectioned (thickness, 4 μm) before HRP-conjugated dUTP staining. Apoptotic cells in the implanted tumors were detected using a TUNEL apoptosis detection kit (Nanjing KeyGen Biotech, KGA7051, China) according to the manufacturer’s instructions. All sections were assessed under the microscope (Nikon, Japan). For each group, the number of apoptotic cells and the total number of cells in five random fields (magnification, ×100) were photographed and counted. The apoptotic index of the cancer cells was calculated using the following formula: Apoptotic index = apoptotic cells/total cells × 100%.

### Statistical analysis

The data were expressed as mean ± standard deviation (SD). Clinicopathological findings were compared using unpaired *t*-tests or Pearson *χ* ^2^ tests with the Social Sciences (SPSS) software version 19.0. Analysis of variance (ANOVA) was used to compare the treated group and control group. *P* < 0.05 was considered to indicate statistical significance.

## Results

### MiR-584-5p is down-regulated in human gastric cancer tissues and cells

**Fig. 1:**
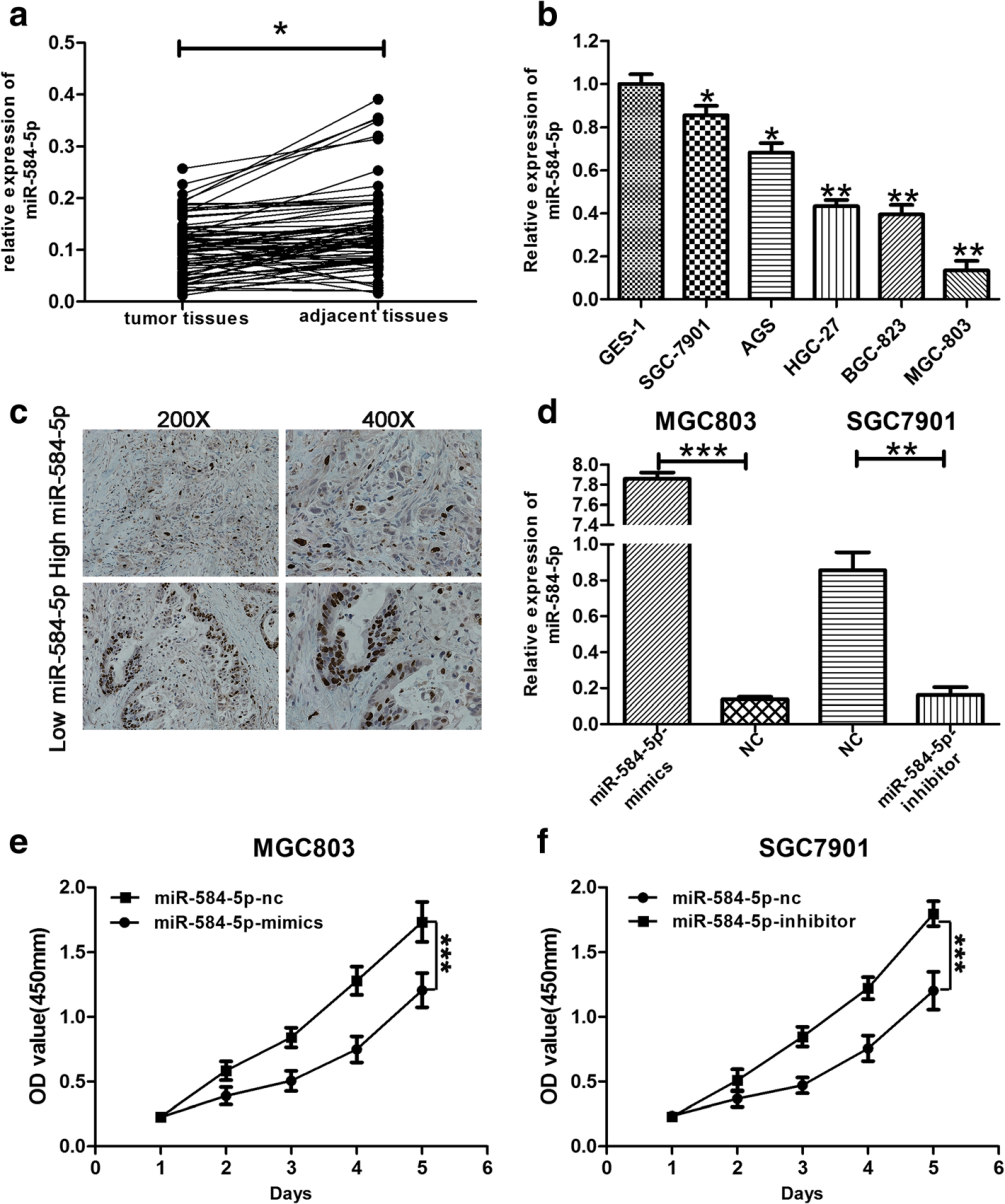
Expression of miR-584-5p in GC tissues, GC cells and transfected cells. **a**. The expression levels of miR-584-5p in 75 pairs of human GC tissues and non-GC tissues were explored using miRNA RT-PCR. **b**. The expression levels of miR-584-5p in GC cells and GES-1 were detected by miRNA RT-PCR. **c**. Immunohistochemical staining against Ki-67 collected from GC patient samples showed a negative correlation between the expression of miR-584-5p and Ki67. **d**. miRNA RT-PCR was used to verify the expression of miR-584-5p in cells transfected with miR-584-5p-mimics and miR-584-5p-inhibitor lentivirus respectively. **e**, **f**. CCK-8 was used to determine the proliferation of GC cells transfected with miR-584-5p-mimics and miR-584-5p-inhibitor lentivirus. OD value between miR-584-5p-mimics or miR-584-5p-inhibitor and corresponding control group was significantly different at *p* < 0.001 by two-way ANOVA. *p < 0.05, **p < 0.01, ***p < 0.001. The data expressed as the mean ± SD

To explore whether miR-584-5p is dysregulated in GC tissues, we collected 75 human GC tissue samples and paired adjacent normal tissues to detect the expression of miR-584-5p by miRNA RT-PCR. As shown in Fig. 1a, miR-584-5p expression was lower in human GC tissues compared with the paired adjacent normal tissues. We further examined miR-584-5p expression in normal gastric mucosa epithelial cells (GES-1) and GC cells lines (HGC27, MGC803, AGS, BGC823, and SGC7901) by miRNA RT-PCR. As shown in Fig. 1b, miR-584-5p expression was lower in the GC cell lines than that in GES-1 cells. In addition, we analyzed the correlation between miR-584-5p expression levels and the clinicopathological characteristics of GC patients (age, gender, tumor size, histology grade, TMN stage, T grade and lymph node metastasis). GC patients were divided into two groups according to miR-584-5p expression levels. GC patients with higher and lower than median expression of miR-584-5p were allocated to the High and Low expression groups, respectively. As shown in Table 1, miR-584-5p expression levels correlated negatively correlated with the tumor size larger than 3 cm. An analysis of the association between miR-584-5p and the proliferation marker, Ki-67, was then performed by staining tumors representative of the High and Low expression groups. As shown in Fig. 1c, the distribution of Ki-67 showed that tumors expressing high miR-584-5p levels were associated with a lower proliferation index compared with the tumors expressing lower miR-584-5p.

**Table 1:** Expression of miRNA-584-5p expression and WWP1 in human gastric cancer according to patients’ clinicopathological characteristics

Characteristics	Number	miR-584-5p expression		*P*-value	WWP1 expression		*P*-value
		High group	Low group		High group	Low group	
Age(years)							
< 60	22	9	13	0.428	12	10	0.561
≥ 60	53	27	26		25	28	
Gender							
Male	34	19	15	0.213	16	18	0.256
Female	41	17	24		14	27	
Size(cm)							
< 3	36	25	11	0.007*	13	23	0.004*
≥ 3	39	15	24		27	12	
Histology grade							
Well-moderately	23	14	9	0.184	12	11	0.630
Poorly-signet	52	23	29		24	28	
Stage							
I/II	26	15	11	0.701	9	17	0.063
III/IV	49	26	23		28	21	
T grade							
T1 + T2	25	15	10	0.191	16	9	0.141
T3 + T4	50	22	28		23	27	
Lymph node metastasis							
Present(N1-N3)	47	26	21	0.179	27	20	0.128
Absent(N0)	28	11	17		11	17	

*p < 0.05 Statistically significant difference

### MiR-584-5p inhibits the proliferation of gastric cancer cells

**Fig. 2:**
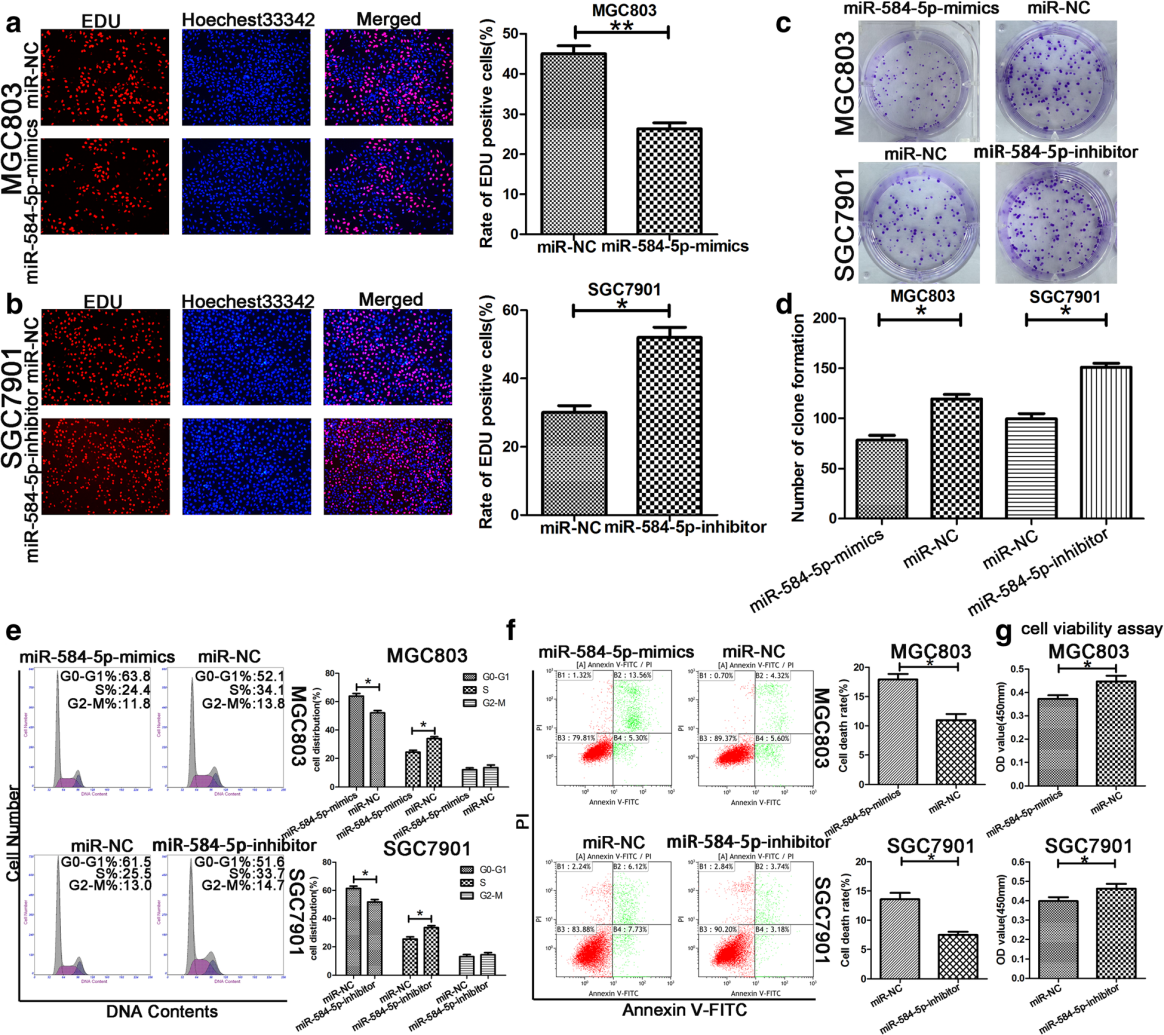
miR-584-5p inhibits cell proliferation and induces cell apoptosis. **a**, **b**. Representative profiles of Edu cell growth in MGC803 cells and SGC7901 cells after transfection with miR-584-5p-mimics and miR-584-5p-inhibitor respectively compared with the control. **c**, **d**. Effects of miR-584-5p alteration on the colony formation of GC cells. **e**. Effects of miR-584-5p alteration on cell cycle distribution of GC cells. **f**. FACS analysis of the effect of miR-584-5p expression alteration on cell apoptosis. **g**. Cell viability assay was explored the effect of miR-584-5p expression alteration on cell apoptosis. *p < 0.05, **p < 0.01, ***p < 0.001. The data expressed as the mean ± SD

Based on the miR-584-5p expression levels in GC cells determined by miRNA RT-PCR, MGC803 and SGC7901 cells were selected for transfection with miR-584-5p-mimic or inhibitor lentivirus constructs to investigate the biological functions of miR-584-5p in GC. The efficiency of transfection of the cell lines (MGC803-mimic, MGC803-NC, SGC7901-inhibitor, SGC7901-NC) was verified by miRNA RT-PCR. As shown in Fig. 1d, miR-584-5p was upregulated significantly in MGC803-mimic and downregulated in SGC7901-inhibitor compared with the levels detected in the control groups. The influence of miR-584-5p on GC cell proliferation was then investigated using the CCK-8 method. The proliferation rate of MGC803 cells transfected with miR-584-5p-mimic was significantly decreased relative to that of the control group. In contrast, SGC7901 cells transfected with the miR-584-5p-inhibitor exhibited a significant increase in proliferation compared with that of the control group (Fig. 1e, f). The EdU incorporation assay was also employed for a more sensitive and specific evaluation of the effect of miR-584-5p on proliferation. As shown in Fig. 2a and b, the number of MGC803 cells incorporating EdU in the miR-584-5p-mimic-treated group was distinctly decreased compared with the number in the control group, while SGC7901 cells transfected with the miR-584-5p-inhibitor revealed a significant increase in cell proliferation compared with that of the control group. In addition, the long-term influence of miR-584-5p on cell proliferation was assessed using the colony formation assay. The results revealed that miR-584-5p overexpression impaired colony formation ability, whereas miR-584-5p knockdown had the opposite effects (Fig. 2c and d). The relevance of cell-cycle interruption in the impaired proliferation induced by miR-584-5p overexpression was analyzed by flow cytometry. As shown in Fig. 2e, MGC803 cells transfected with miR-584-5p-mimic exhibited a significant increase in the percentage of cells in the G0/G1 phase, whereas the opposite trend was observed in SGC7901 cells transfected with the miR-584-5p-inhibitor. These observations indicated that miR-584-5p overexpression induced cell-cycle arrest in the G0/G1 phase, while miR-584-5p knockdown promoted GC cell proliferation.

### MiR-584-5p induces cell apoptosis of gastric cancer cells

Apoptosis is an important cause of tumor suppression. Flow cytometric analysis of apoptosis was performed to confirm the assumption that miR-584-5p functions as a candidate tumor suppressor gene in GC. MGC803 cells transfected with miR-584-5p-mimic exhibited a higher rate of apoptosis compared with that of the control group, while the opposite trend was observed in SGC7901 cells transfected with miR-584-5p-inhibitor (Fig. 2f). The results of CCK-8 cell viability assays were consistent with the results of the flow cytometric analysis (Fig. 2g). Based on these findings, we suggested that miR-584-5p accelerates the progression of GC cell apoptosis.

### WWP1 3′-UTR is a putative target of miR-584-5p

TargetScan (http://www.targetscan.org/), PicTar (http://pictar.mdc-berlin.de/) and miRanda (http://www.microrna.org/microrna/home.do) were employed to predict target genes of miR-584-5p. Bioinformatics analysis revealed WWP1, a well-studied tumor oncogene associated with many malignancies, to be a potential target of miR-584-5p.

### WWP1 is frequently upregulated in GC tissues and cells

**Fig. 3:**
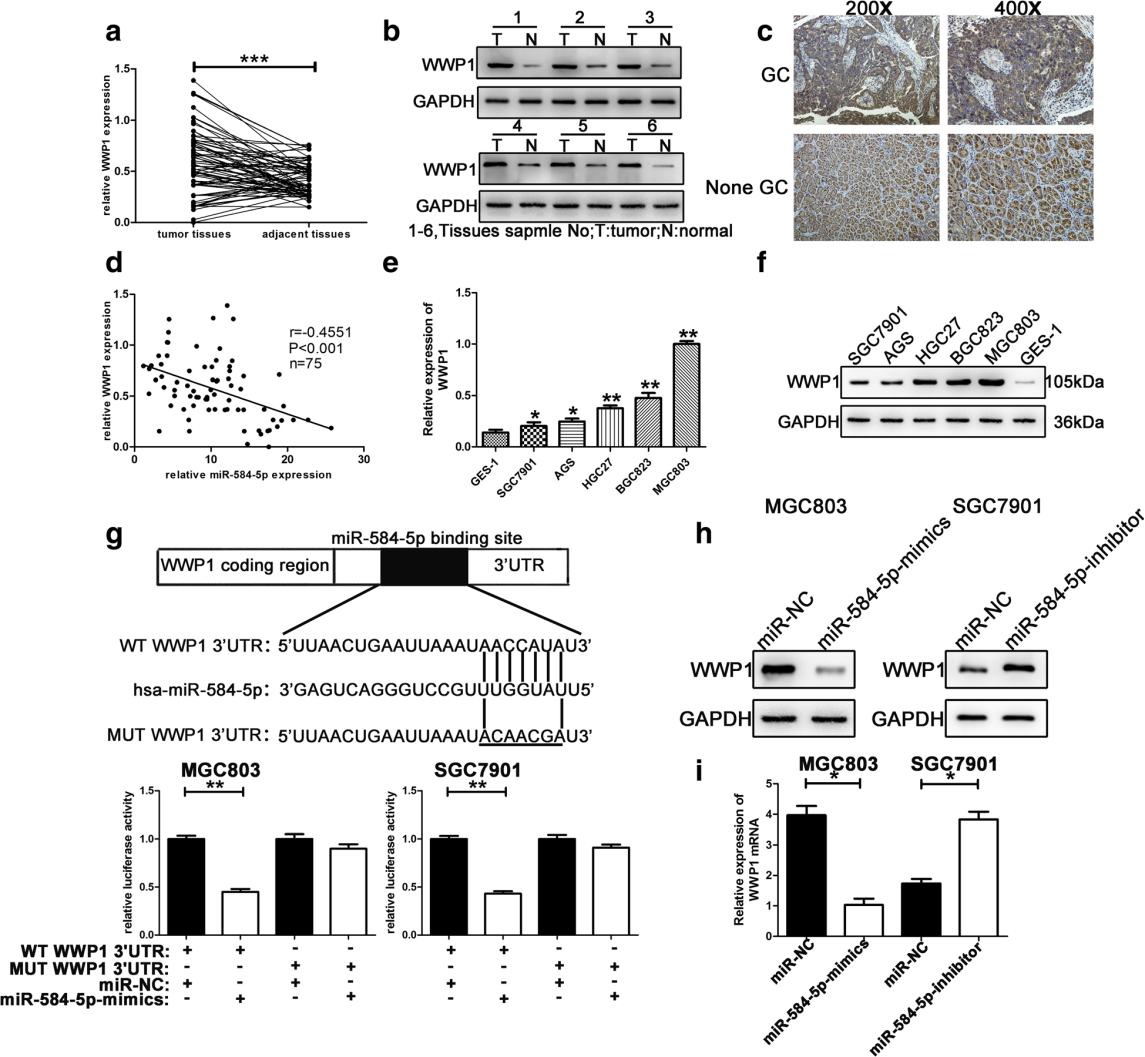
WWP1 was up-regulated in GC tissues and cells. WWP1 was proved to be the potential target gene of miR-584-5p. **a**. The expression level of WWP1 was detected in 75 pairs of human GC tissues and non-GC tissues by qRT-PCR. **b**. WWP1 protein level was determined using Western blot in six paired of GC tissues. GAPDH was used as the internal reference. **c**. The expression level of WWP1 protein in GC tissues and adjacent normal tissues was determined by immunohistochemistry staining. **d**. Negative correlation between the expression levels of miR-584-5p and WWP1 in GC specimens. **e**, **f**. The expression levels of WWP1 in GC cells and GES-1 were detected using qRT-PCR and Western blot. **g**. Luciferase reporter assay was conducted to verify that miR-584-5p directly bound to the 3’-UTR region of WWP1. Luciferase activity was analyzed in cells co-transfcted with miR-584-5p-mimics or negative control with pGL3-WWP1-WT or pGL3-WWP1-MUT. **h**. The expression level of WWP1 protein in GC cells after miR-584-5p expression alteration by Western blot. **i**. The expression level of WWP1 mRNA in GC cells after miR-584-5p expression alteration by qRT-PCR. *p < 0.05, **p < 0.01, ***p < 0.001. The data expressed as the mean ± SD

To evaluate the pattern of miR-584-5p and WWP1 expression in GC tissues, we performed qRT-PCR analysis of WWP1 expression in 75 paired human GC specimens and adjacent normal tissues. As shown in Fig. 3a, compared with matched adjacent normal tissues, WWP1 expression was significantly elevated in GCs. We then explored WWP1 protein expression in six randomly selected pairs of GC specimens and adjacent normal tissues samples by Western blot. As shown in Fig. 3b, WWP1 expression was higher in GC tissues than that in the paired adjacent normal tissues. Similarly, immunohistochemical investigations revealed elevated WWP1 expression in GC tissues compared with that in the paired adjacent normal tissues (Fig. 3c). We also found that miR-584-5p expression levels were inversely associated with WWP1 expression (Fig. 3d). Furthermore, we analyzed the correlation between WWP1 expression levels and clinicopathological features. As shown in Table 1, WWP1 expression levels showed a highly positive correlation with size in tumors larger than 3 cm. Moreover, qRT-PCR analysis revealed higher WWP1 expression levels in GC cell lines compared with those in GES-1 cells (Fig. 3e). In accordance with the analysis of GC tissues, Western blot analysis showed an inverse correlation between WWP1 expression and miR-584-5p expression in GC cell lines and GES-1 cells (Fig. 3f). These results indicated that WWP1 is a putative target gene of miR-584-5p in GC, which is consistent with our hypothesis.

### MiR-584-5p interacts directly with a putative binding site of WWP1-3′-UTR

The role of WWP1 as a direct target of miR-584-5p was further validated in dual luciferase reporter assays. Mutant-type (MUT) and wild-type (WT) WWP1 3′-UTR sequences (the former containing site-directed mutations in the putative miR-584-5p target sites) were cloned into reporter plasmids (Fig. 3g). Co-transfection of both MGC803 and SGC7901 cells with miR-584-5p-mimic and the pGL3-WT-WWP1 3′-UTR resulted in significantly decreased luciferase activity compared with that in the control group (Fig. 3e). Nevertheless, there was no significant reduction in luciferase activity following co-transfection with pGL3-MUT-WWP1 3′-UTR and miR-584-5p-mimic. These results indicated that the 3′-UTR of WWP1 is targeted by miR-584-5p and that the mutations in this sequence abolish this interaction.

### MiR-584-5p suppresses WWP1 protein expression by mRNA degradation

WWP1 protein levels were then analyzed by Western blot to investigate the mechanism by which miR-584-5p reduced WWP1 expression. WWP1 protein levels were significantly reduced in MGC803 cells, which express high levels of endogenous WWP1, after transfection with miR-584-5p-mimic. In contrast, WWP1 protein levels were elevated in SGC7901 cells, which express low levels of endogenous WWP1, after transfection with the miR-584-5p-inhibitor (Fig. 3h). Furthermore, compared with the control, evident changes were observed in WWP1 mRNA levels following transfection of GC cells with the miR-584-5p-mimic or miR-584-5p-inhibitor (Fig. 3i). This finding suggested that miR-584-5p suppresses WWP1 protein expression by degrading the corresponding mRNA.

### MiR-584-5p inhibits proliferation and induces apoptosis in GC cells by targeting WWP1

**Fig. 4:**
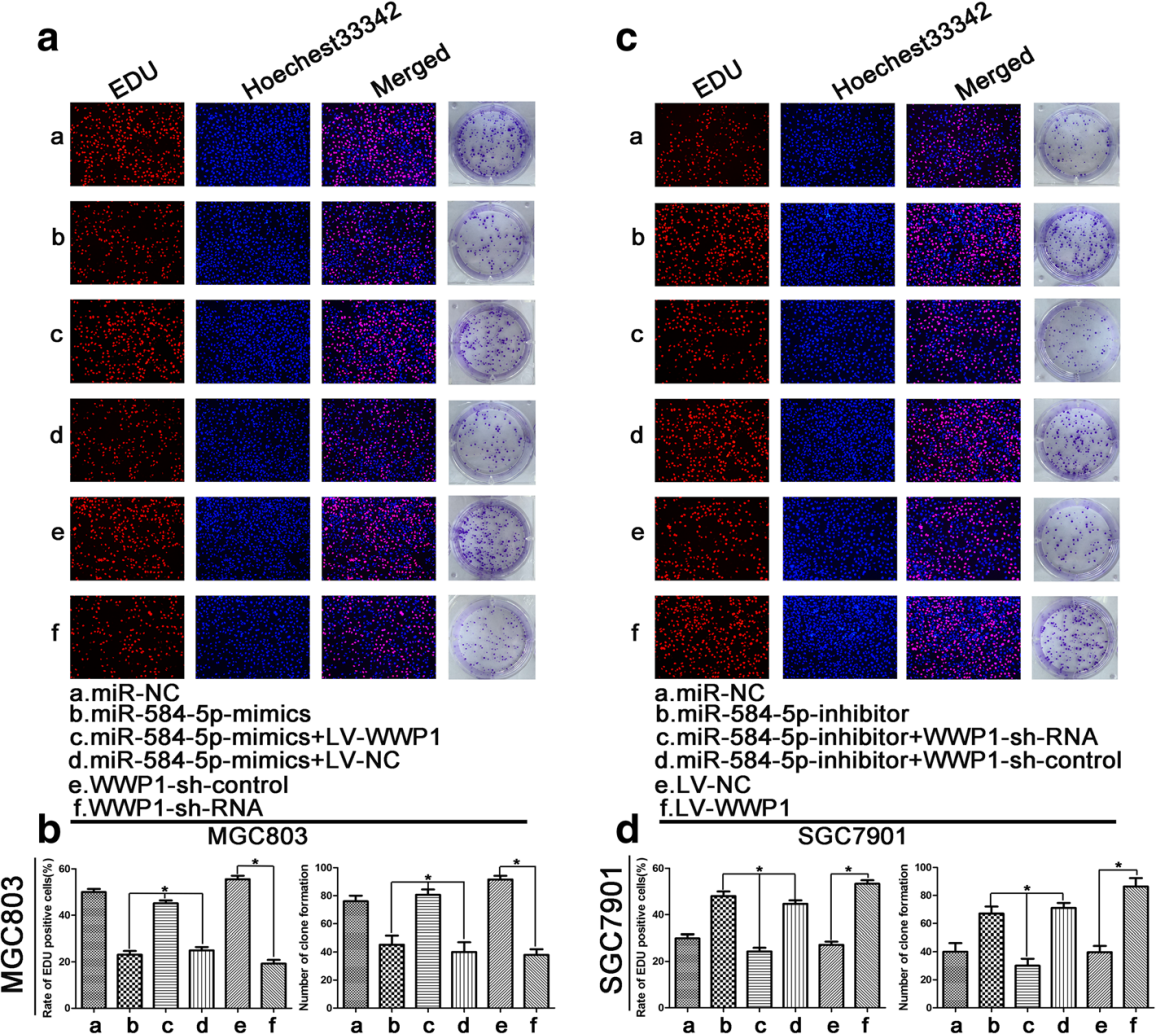
The roles of miR-584-5p and WWP1 in the regulation of gastric cancer cell proliferation and apoptosis. **a**. miR-584-5p up-regulation or WWP1-shRNA inhibits proliferation and induces apoptosis using the EdU assay and colony formation assay. The rescue experiments for miR-584-5p overexpression were performed by ectopic expression of WWP1 without its 3’-UTR in MGC803 cells. **c**. Similar rescue experiments for miR-584-5p silencing was performed by downregulation of WWP1 in SGC7901 cells. **b**, **d**. The data came from at least three independent experiments. A representative data set is displayed as mean ± SD values. *p < 0.05, **p < 0.01, ***p < 0.001

**Fig. 5:**
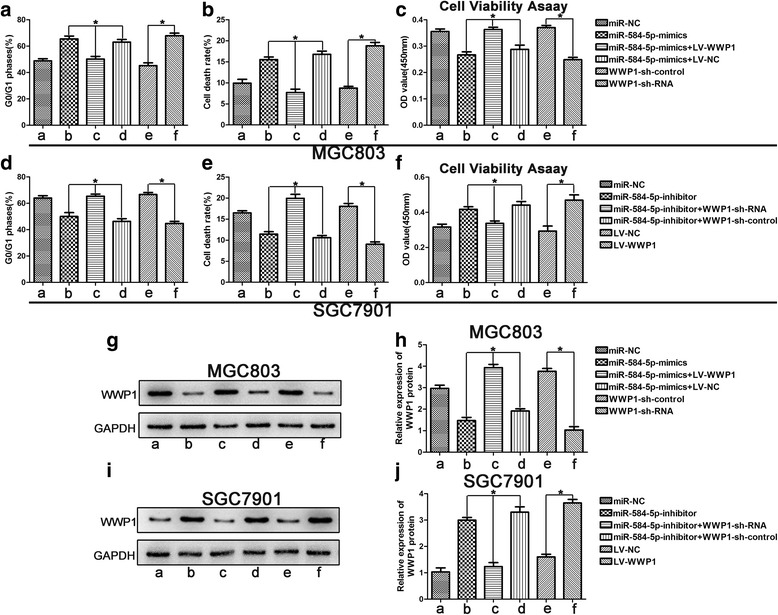
The roles of miR-584-5p and WWP1 in the regulation of gastric cancer cell proliferation and apoptosis. **a**-**c**. miR-584-5p up-regulation or WWP1-shRNA inhibits proliferation and induces apoptosis using the cell cycle assay, flow cytometry and cell viability assay. The rescue experiments for miR-584-5p overexpression were performed by ectopic expression of WWP1 without its 3’-UTR in MGC803 cells. **d**-**f**). Similar rescue experiments for miR-584-5p silencing was performed by downregulation of WWP1 in SGC7901 cells. **g**-**j**. Western blot was used to verify the expression of WWP1. The data came from at least three independent experiments. A representative data set is displayed as mean ± SD values. *p < 0.05, **p < 0.01, ***p < 0.001

We demonstrated that ectopic expression of miR-584-5p inhibited proliferation and induced apoptosis in GC cells and inhibited WWP1 protein expression by degrading WWP1 mRNA. In contrast, miR-584-5p knockdown had the opposite effect, leading to enhanced WWP1 protein expression. To further verify that the effects of miR-584-5p on proliferation and apoptosis in GC cells were mediated by regulation of WWP1, we silenced endogenous WWP1 expression in MGC803 cells by using shRNA technology. As shown in Figs. 4a, 5a, b, c, (e and f), knockdown of endogenous WWP1 inhibited MGC803 cell proliferation, while apoptosis was promoted, which was consistent with the effects of miR-584-5p upregulation (Figs. 4a, 5a, b, c, [a and b]). WWP1 overexpression in SGC7901 cells was induced by transfection with a WWP1 lentivirus. As shown in Figs. 4c, 5d, e, f, (e and f). WWP1 overexpression promoted SGC7901 cell proliferation and inhibited apoptosis, which was consistent with the effects of miR-584-5p downregulation (Figs. 4c, 5d, e, f, [a and b]). Subsequently, we investigated the ability of WWP1 to counteract the effects of miR-584-5p overexpression. The vector LV-WWP1, which contained only the WWP1 coding sequence, was constructed to allow WWP1 expression in the absence of the miR-584-5p target sequence. MGC803 cells were co-transfected with miR-584-mimic and either LV-WWP1 or LV-NC. The results demonstrated that ectopic WWP1 expression effectively reversed the suppression of proliferation and promotion of apoptosis induced by miR-584-5p overexpression (Figs. 4a, 5a, b, c, [c and d]). Similarly, the effects of miR-584-5p knockdown were counteracted by WWP1 downregulation in SGC-7901 cells (Figs. 4c, 5d, e, f, [c and d]). WWP1 protein expression levels in transfected cells were confirmed by Western blot analysis (Fig. 5g–j). These findings are consistent with our hypothesis that miR-584-5p inhibits GC cell proliferation and induces apoptosis by targeting WWP1 directly.

### MiR-584-5p inhibits the tumorigenicity in vivo

**Fig. 6:**
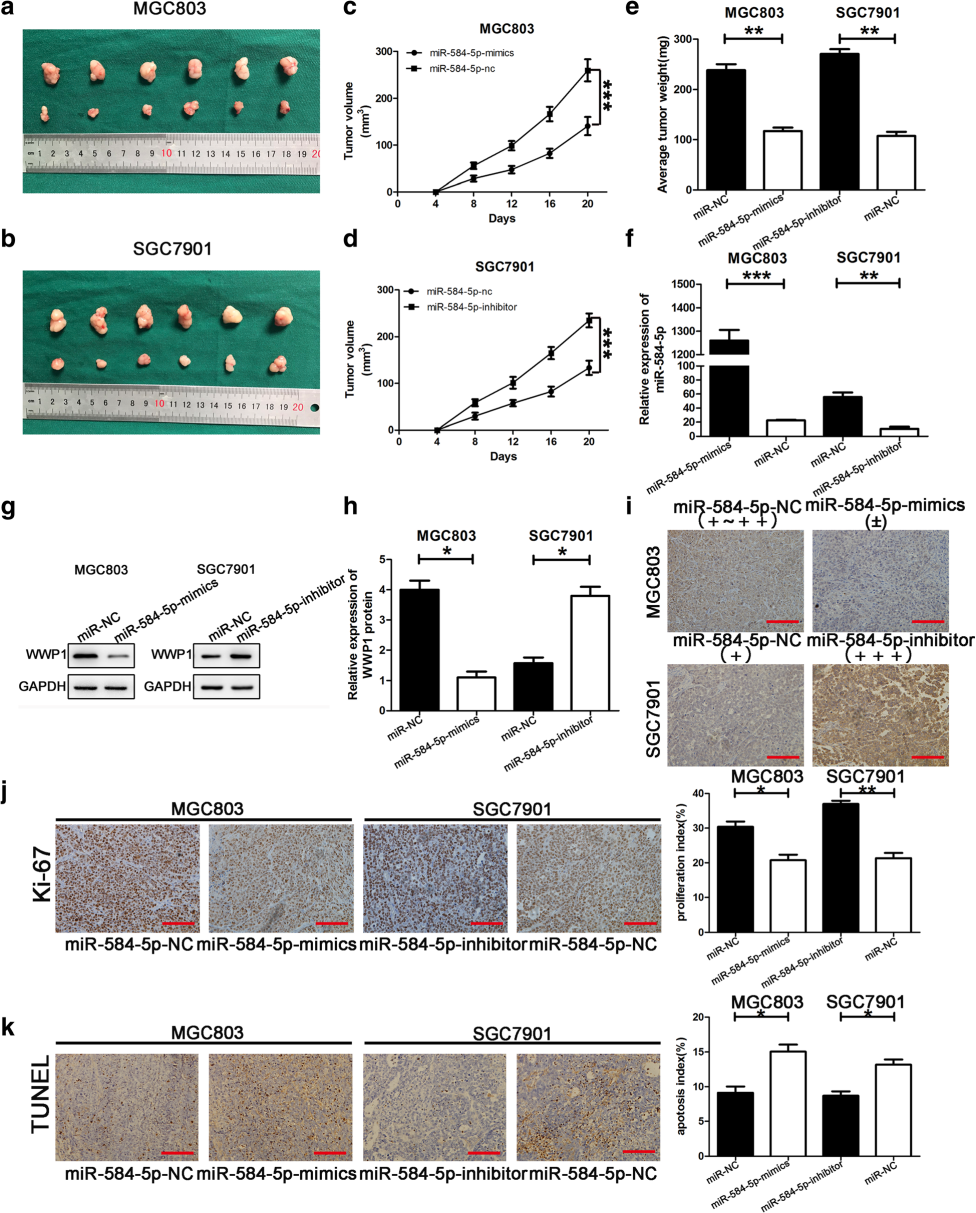
miR-584-5p inhibits tumorigenicity in vivo. **a**, **b**. Photographs of tumors obtained from the different groups of nude mice transfected with miR-584-5p-mimics and miR-584-5p-inhibitor, respectively. **c**, **d**. Growth curve of tumor volumes were calculated. Date are shown as mean ± SD. Tumor volumes between miR-584-5p-mimics or miR-584-5p-inhibitor and corresponding control group was significantly different at *p* < 0.001 by two-way ANOVA. **e**. Tumor weight were calculated. Date are shown as mean ± SD. **f**, **g**, **h**, **i**. The expression levels of miR-584-5p and WWP1 protein in the implanted tumors that were transfected with miR-584-5p-mimics and miR-584-5p-inhibitor were explored by miRNA RT–PCR,qRT-PCR,Western blot and immunohistochemistry staining. **j**, **k**. Immunohistochemical staining against Ki-67 and TUNEL assay were used to determine the effects of miR-584-5p expression alteration on cell proliferation and apoptosis in the samples collected from nude mice. Scale bar is 100 μm. *p < 0.05, **p < 0.01, ***p < 0.001. The data expressed as the mean ± SD

To investigate the effects of miR-584-5p on tumorigenicity in vivo, cells transfected with the vectors mentioned above were injected into the flanks of nude mice to generate tumors ectopically. As shown in Fig. 6a–e, the tumor volume and weight in the miR-584-5p-mimic group were decreased compared with those in the miR-584-5p-NC group (MGC803 cells). Conversely, the tumor volume and weight in the miR-584-5p-inhibitor group were increased compared with those in the miR-584-5p-NC group (SGC7901 cells). Furthermore, miRNA RT-PCR analysis showed elevated miR-584-5p expression in the miR-584-5p-mimic-treated group, while the opposite pattern of expression was observed in miR-584-5p-inhibitor-treated group (Fig. 6f). Furthermore, Western blot and immunohistochemical analyses of the implanted tumors in mice revealed significant downregulation of WWP1 protein expression in the miR-874-mimic-transfected group compared with that in the control group. In contrast, WWP1 protein expression was significantly upregulated in the miR-584-5p-inhibitor-transfected group compared with that in the control group (Fig. 6g–i). Subsequently, Ki-67 staining and TUNEL assays were used to further verify that miR-584-5p suppressed tumorigenicity. The distribution of Ki-67 revealed a reduced proliferation index in the miR-584-5p-mimic-transfected group compared with that of the control group, while the opposite trend was observed in the miR-584-5p-inhibitor-transfected group. TUNEL assays revealed a dramatically higher apoptosis index in the miR-584-5p-mimic-transfected tumors compared with that in the control group, thus demonstrating decreased cell viability. In contrast, a dramatically lower apoptosis index in was observed in the miR-584-5p-inhibitor-transfected group (Fig. 6j, k).

### MiR-584-5p promotes senescence by targeting WWP1

**Fig. 7:**
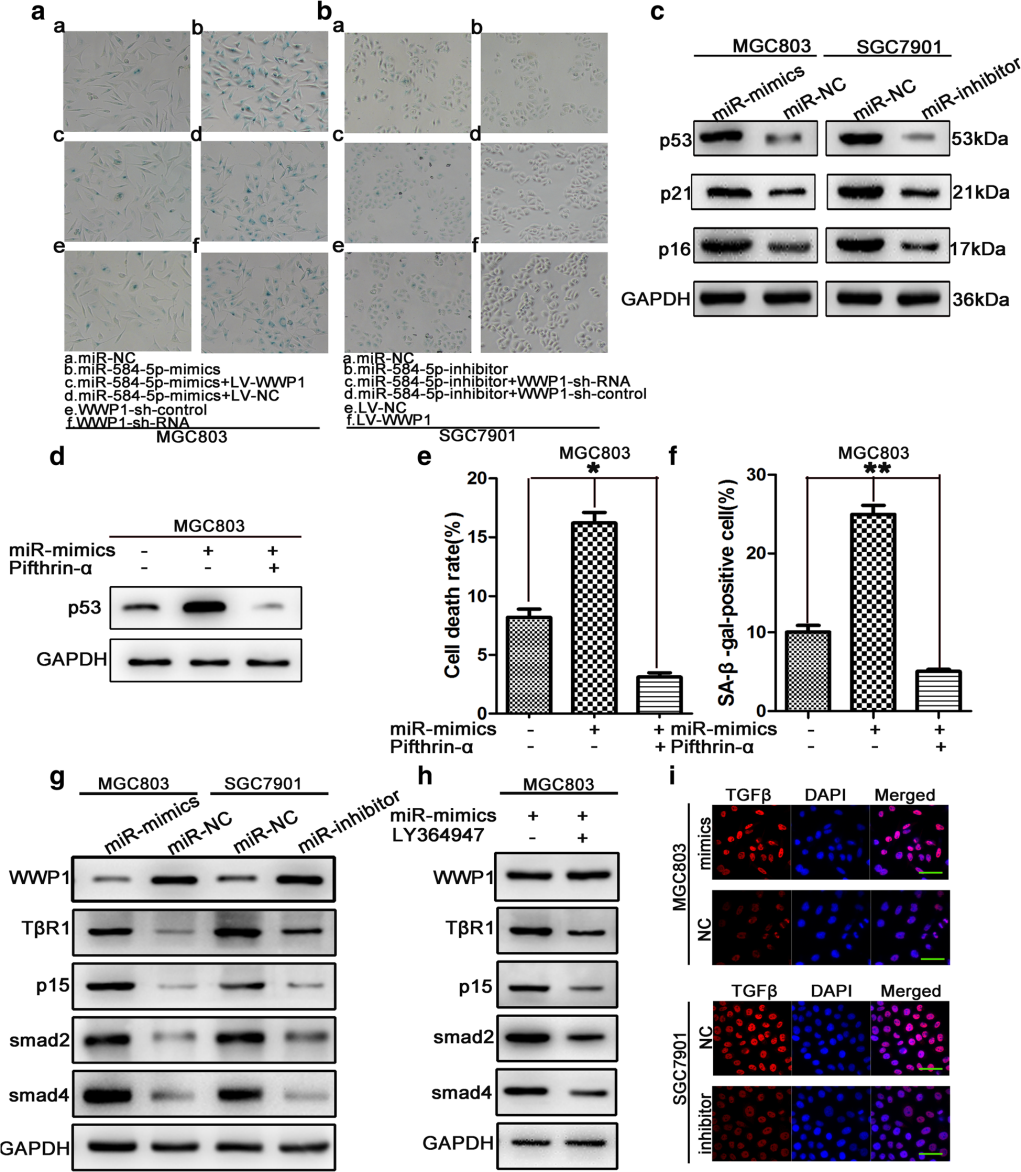
miR-584-5p promoted cellular senescence and negatively regulated TGFβ signaling pathway via downregulation of WWP1. **a**. miR-584-5p up-regulation or WWP1-shRNA promoted cellular senescence by detecting SA-β-gal activity. The rescue experiments for miR-584-5p overexpression were performed by ectopic expression of WWP1 without its 3’-UTR in MGC803 cells. **b**. Similar rescue experiments for miR-584-5p silencing was performed by down-regulation of WWP1 in SGC7901 cells. **c**. The expression levels of senescence-related proteins were evaluated by Western blot. **d**. Expression of p53 signifcantly increased by miR-584-5p was ameliorated by the p53 inhibitor pifthrin-α. **e**. Treatment of pifthrin-α signifcantly receded miR-584-5p induced apoptosis of the MGC803 cells which was measured in a flow cytometer. **f**. Positive SA-β-Gal stained cells induced by miR-584-5p was confined by treatment of pifthrin-α. **h**. Levels of key proteins involved in TGFβ signaling by western blot. **i**. Levels of key proteins involved in TGFβ signaling in MGC803 cells treated with LY364947 were measured by western blot. **j**. The expression level of TGFβ was determined in MGC803 and SGC7901 by immunofluorescence assays. Scale bar is 25 μm. *p < 0.05, **p < 0.01, ***p < 0.001. The data expressed as the mean ± SD

Considering that WWP1 overexpression accelerated tumor cell proliferation and survival and delayed cellular senescence, we evaluated the role of miR-584-5p in the regulation of GC cellular senescence by detection of SA-β-gal staining, which is considered a specific senescence marker. As shown in Fig. 7a, b, (a and b). The miR-584-5p-mimic-transfected cells showed strong blue SA-β-gal staining, while only scattered SA-β-gal-positive cells were detected in miR-584-5p-inhibitor-transfected group compared with the corresponding control cells. In experiments to further confirm that miR-584-5p promotes senescence by regulating WWP1, we observed that knockdown of endogenous WWP1 resulted in strong blue SA-β-gal staining compared with the control, while WWP1 overexpression showed only weak SA-β-gal staining. These observations were consistent with the effects of miR-584-5p upregulation in MGC803 cells and downregulation of miR-584-5p in SGC7901 cells (Fig. 7a, b [e and f]). Intriguingly, co-transfection of MGC803 cells with miR-584-mimic and either LV-WWP1 or LV-NC showed that ectopic expression of WWP1 effectively reversed the promotion of cellular senescence resulting from miR-584-5p overexpression. Similarly, the effects of miR-584-5p knockdown in SGC7901 were counteracted by WWP1 downregulation (Fig. 7a, b, [c and d]). To further confirm the effects of miR-584-5p on cellular senescence, the expression levels of several senescence markers were evaluated by Western blot. As shown in Fig. 7c, the expression of p53, p21 and p16 was enhanced after miR-584-5p-mimic-transfection. Conversely, the expression of p53, p21 and p16 was reduced after miR-584-5p-inhibitor transfection. These findings demonstrated that miR-584-5p overexpression promotes cellular senescence by silencing WWP1 expression.

### p53 is involved in miR-584-5p-induced induction of apoptosis and senescence

The main mechanisms by which p53 functions as a tumor suppression are p53-mediated apoptosis and senescence. To determine the role of p53 in miR-584-5p-induced induction of apoptosis and senescence, we inhibited p53 expression in MGC803 cells by treatment with the specific inhibitor, pifithrin-α for 24 h (Fig. 7d). As shown in Fig. 7e, pifithrin-α treatment significantly reduced miR-584-5p-induced apoptosis of the MGC803 cells, indicating the involvement of p53 in the inhibitory effects of miR-584-5p. The induction of SA-β-Gal positively stained cells by miR-584-5p was also reduced by treatment of pifithrin-α (Fig. 7f), indicating the involvement of p53 in miR-584-5p-induced cellular senescence.

### MiR-584-5p activates the TGFβ signaling pathway by downregulation of WWP1

Having shown that miR-584-5p promoted proliferation and induced apoptosis of GC cells, we investigated the possibility that these effects are mediated partially by activation of the TGFβ signaling pathway following WWP1 downregulation. Western blot analysis of the expression of WWP1 and key proteins involved in TGFβ signaling pathway showed that the protein levels of TβR1, Smad2, Smad4, and the CDK inhibitor p15 (a downstream target of TGFβ) were increased after miR-584-5p-mimic transfection, while the opposite effects were observed after miR-584-5p-inhibitor transfection (Fig. 7h). To confirm that TGFβ signaling pathway activation was mediated by miR-584-5p, MGC803 cells transfected with miR-584-5p-mimic were treated for 24 h with the TGF-β signaling pathway inhibitor, LY364947. No significant difference was observed in WWP1 expression between the two groups. However, the expression of TβR1, Smad2, Smad4, and p15 was decreased in LY364947 treated cells compared with that of the control group (Fig. 7i). Furthermore, immunofluorescence assays revealed a dramatically positive correlation between the levels of miR-584-5p and TGFβ (Fig. 7g). Taken together, our results suggested that the effects of miR-584-5p are mediated via WWP1 downregulation and subsequent activation of the TGFβ signaling pathway.

## Discussion

Aberrant expression of miRNAs plays a vital role in the initiation and development of cancer [20, 21]. MiRNAs have shown to act as either oncogenic factors or tumor suppressors, depending on the specific functions of the targeted mRNA. Decreased expression of tumor suppressor miRNAs results in increased oncogene translation, which contributes to the development and progression of cancer. Similar effects are caused by increased expression of oncogenic miRNAs leads to inhibition of tumor suppressor genes [22].

Although miR-584-5p has been identified as a tumor suppressor and found to be downregulated in certain malignancies [11–14], the expression pattern and functions of miR-584-5p in cancer remain to be fully elucidated. Therefore, further investigation of the role of miR-584-5p in cancer development is warranted. In this study, we found that miR-584-5p expression was dramatically lower in GC tissues and cell lines compared with paired adjacent non-GC tissues and GES-1 cells. We explored the role of miR-584-5p in proliferation and apoptosis both in vitro and in vivo. Our results showed that miR-584-5p overexpression suppressed proliferation and promoted apoptosis both in vitro and in vivo, while the converse effects were observed following miR-584-5p knockdown. These results indicated the importance of miR-584-5p downregulation in the development and progression of GC.

To clarify the mechanisms underlying the effects of miR-584-5p on proliferation and apoptosis, we predicted putative targets of miR-584-5p in GC cells by bioinformatics analysis. Among the candidate target genes, we concentrated on WWP1, which is located on chromosome 8q21 and is an E3 ubiquitin ligase first identified because of its specific WW domain [23]. WWP1 consists of an N-terminal C2 domain, a C-terminal catalytic HECT domain for ubiquitin transfer, and four tandem WW domains for substrate binding [24]. It has been recognized as a key regulator of a variety of human cancers, infectious diseases, neurological diseases, and aging [15]. Accumulating evidence has demonstrated the involvement of WWP1 in the regulation of various biological processes, such as protein trafficking and degradation, and cellular senescence, as well as signal transduction and transcription. Studies have also revealed that WWP1 plays vital roles in the initiation, development, progression, and prognosis of numerous malignancies, including prostate cancer, breast carcinoma, hepatocellular carcinoma and GC [25–28]. In the present study, stepwise investigations revealed differential expression of WWP1 between GC tissues and paired adjacent non-GC tissues, as well as in GC cell lines and normal epithelial cells. WWP1 expression was also found to be associated with tumor size exceeding 3 cm in GC patients. Furthermore, miR-584-5p negatively regulated WWP1 at the translational level by binding to a specific target site within the 3′-UTR, which was further confirmed in luciferase reporter assays. Overexpression of miR-584-5p in human GC cell lines inhibited WWP1 by degrading WWP1 mRNA, and ectopic expression of WWP1 significantly reversed the effects of the suppression of proliferation and promotion of apoptosis caused by miR-584-5p overexpression. In addition, shRNA-mediated silencing of WWP1 impaired the promotion of proliferation and suppression of apoptosis caused by miR-584-5p knockdown. Taken together, the results of our study suggest that the inhibitory effects of miR-584-5p on GC are mediated by downregulation of WWP1.

Cancer as a disease is related to immortal cells, and induction of cellular senescence results in tumor regression due to impaired tumor cell proliferation. Many studies have shown that miRNAs are relevant to cellular senescence. Downregulation of the miR-130b–301b cluster impairs cellular senescence in prostate cancer [29], while miR-494-3p increases the radiosensitivity of oral squamous cell carcinoma cells through the induction of cellular senescence caused by the downregulation of Bmi1 [30]. Restoration of miR-137 expression inhibited proliferation and promoted senescence of pancreatic cancer cells [31]. Cellular senescence is a state of irreversible proliferative arrest that results in a senescent phenotype, which is characterized by increased SA-β-gal activity, inhibited cell proliferation and cell-cycle arrest. Furthermore, many genes such as p53, p21, p16, are considered to be senescence markers or have been associated with replicative senescence [32–35]. p53 plays a vital role in cellular senescence and is is the main transcription factor involved in the regulation of p21 expression. p21 and p16 are the most significant signaling factors that regulate senescence. Both are cyclin-dependent-kinase inhibitors, and play different roles in the initiation and maintenance of cell-cycle arrest in senescence [36]. In this study, we showed that miR-584-5p promotes cellular senescence by downregulation of WWP1.

Although the molecular mechanism of p53-mediated tumor suppression is not entirely clear, it is widely accepted that tumor growth is suppressed by p53-mediated cell-cycle arrest, apoptotic cell death, and cellular senescence. In cell-based experiments, artificial activation of p53 contributed to tumor-specific apoptosis and senescence. These findings have resulted in the development of drugs that restore p53 activity [37]. The role of p53 in miR-584-5p-induced induction of apoptosis and senescence was investigated using the selective p53 inhibitor, pifithrin-α. Our results revealed that p53 mediates miR-584-5p-induced apoptosis and senescence.

TGFβ inhibits the growth of many types of cells, and various cancer cell lines have been shown to be resistant to the inhibitory effects of TGFβ on growth. Therefore, inactivation of the TGFβ signaling pathway or enhanced expression of the signaling inhibitors may facilitate tumor progression. Smad7 and Smurf2 have been identified as TGFβ signaling inhibitors that are overexpressed in some types of cancer. Recently, WWP1 was shown to modulate various signaling pathways and act as a Smad7-binding protein to inhibit TGFβ signaling [16]. Therefore, we also explored the involvement of the TGFβ signaling pathway in the effects of alterations in miR-584-5p expression in GC. We found that miR-584-5p activated the TGFβ signaling pathway via downregulation of WWP1, thus, releasing its negative regulatory effects on TGFβ signaling pathway, and leading to suppression of proliferation and promoted apoptosis. However, the results of this study do not rule out the possibility that other signaling pathways may also be affected by miR-584-5p. Furthermore, the relationship between miR-584-5p and clinical outcome requires further investigation.

## Conclusion

Our study demonstrates that miR-584-5p is associated with tumor size and that miR-584-5p suppresses GC development and progression in a WWP1-dependent manner. MiR-584-5p is also confirmed as an important member of the senescence-regulatory group of miRNAs. We identified the possible mechanisms by which miR-584-5p regulates cell proliferation, apoptosis and senescence, and this information is important in developing strategies to control gastric cancer progression by eliminating cancer cells. Furthermore, the association between miR-584-5p expression and tumor size, as well as its tumor suppressor function suggest the potential of miR-584-5p as a novel therapeutic target for GC.

## Abbreviations

BSA: bull serum albumin
CCK-8: cell counting kit 8
DAPI: 4',6-diamidino-2-phenylindole
FBS: fetal bovine serum
GC: gastric cancer
PBS: phosphate buffered solution
PCR: polymerase chain reaction
TUNEL: terminal deoxynucleotidyl transferase-mediated dUTP nick end labeling
WWP1: WW domain-containing E3 ubiquitin protein ligase 1
